# Acute Idiopathic Scrotal Edema

**DOI:** 10.1155/2013/829345

**Published:** 2013-11-13

**Authors:** Micheál Breen, Kevin Murphy, Jeanne Chow, Eamon Kiely, Kevin O'Regan

**Affiliations:** ^1^Department of Radiology, Cork University Hospital, Wilton, Cork, Ireland; ^2^Department of Radiology, Boston Children's Hospital, 300 Longwood Avenue, Boston, MA 02115, USA; ^3^Department of Urology, Cork University Hospital, Wilton, Cork, Ireland

## Abstract

We report a case of acute idiopathic scrotal edema (AISE) in a 4-year-old boy who presented with acute scrotal pain and erythema. The clinical features, ultrasound appearance, and natural history of this rare diagnosis are reviewed. In this report, we highlight the importance of good ultrasound technique in differentiating the etiology of the acute scrotum and demonstrate the color Doppler “Fountain Sign” that is highly suggestive of AISE.

## 1. Introduction

Acute idiopathic scrotal edema (AISE) is a benign, self-limiting condition which is a rare cause of acute scrotal erythema. It is more common in the pediatric population than in adults. AISE is a difficult but important diagnosis, as correctly identifying it can avoid unnecessary surgical exploration of the scrotum. We present a case of AISE in a 4-year-old boy confirmed following ultrasound and surgical exploration and highlighting the clinical and sonographic features which in retrospect were indicative of the diagnosis. 

## 2. Presentation of Case

**Figure 1:**
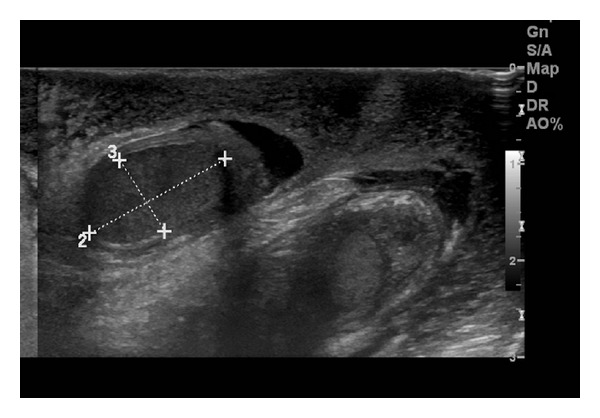
Ultrasound showing marked edematous scrotal skin thickening, small bilateral hydroceles, and normal appearing testes.

**Figure 2:**
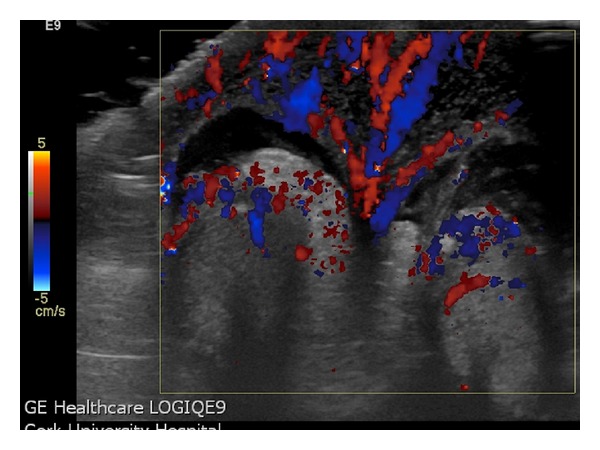
Transverse color Doppler image showing the “Fountain Sign” of increased blood flow in the edematous scrotal skin.

A four-year-old boy was referred to the emergency department from his primary care physician with a four-hour history of bilateral scrotal pain, swelling, and redness. The patient had no medical or surgical history of note. On examination, the scrotum was diffusely erythematous with erythema extending to the perineum. The left hemiscrotum was enlarged and exquisitely tender to palpation. The right hemiscrotum was of normal size and mildly tender in comparison. An ultrasound was organized which showed marked bilateral scrotal skin thickening and small bilateral hydroceles (Figure 1). There was marked increased color flow seen throughout the scrotal skin bilaterally (Figure 2). Both testes and epidydimi were morphologically normal, however; vascular flow was not demonstrated in the testes on color or Doppler interrogation. The child proceeded to surgical exploration which confirmed scrotal wall edema and normal epidydimi and testes bilaterally. A bilateral Jaboulay orchidopexy was performed. The child made an uneventful postoperative recovery with complete resolution of his symptoms. 

## 3. Discussion

The acute scrotum is a diagnostic challenge, both in the pediatric and adult setting. Epididymitis and testicular torsion are the most common causes of acute scrotal pain, especially in adolescents. Torsion of the testicular appendages has a higher incidence in the prepubertal age group [1].

AISE is often a diagnosis of exclusion. It was first described by Qvist in 1959 who reported a prevalence of 20% [2]. It is characterised by marked edema of the skin and dartos fascia without involvement of the deeper layers, testes, or epidydimi [3].

The exact etiology of AISE is unclear. It has been hypothesized that it represents a hypersensitivity reaction related to a variant of angioneurotic edema [4, 5]. It has been associated with eosinophilia, with a 66.7% incidence in one case series [3].

Swelling and erythema in the scrotal wall is characteristic [2], but the condition is not universally painful. AISE can be unilateral or bilateral and extension of redness to the perineum or inguinal region is seen in half of cases [3].

Ultrasound is the imaging modality of choice in the investigation of the acute scrotum. Thickening and edema of the scrotal wall, hypervascularity of the scrotum, and normal appearance of the testes are considered specific for the condition [3, 6].

Geiger has described the “Fountain Sign,” a novel finding on color Doppler interrogation which is highly suggestive of the diagnosis. The “Fountain” depicted on transverse imaging of the scrotum (Figure 2) is due to marked increased hypervascularity in the scrotal wall which derives its blood supply from branches of the deep external pudendal and internal pudendal arteries via the anterior and posterior sacral arteries [7].

Other sonographic findings described in AISE include mild reactive hydrocele and enlarged, hypervascular inguinal lymph nodes [3].

The differential diagnosis for AISE includes other causes of acute scrotum such as epididymitis, testicular torsion, torsion of the testicular, and epididymal appendages and other conditions such as hydrocele and inguinal hernia. Lymphatic malformations of the scrotum can also present with bilateral scrotal pain and hydrocele.

Ultrasound has characteristic findings in each of these diagnoses. Testicular torsion is characterized by hypovascularity of the affected testis, high resistance arterial blood flow as demonstrated by increased resistive indices on Doppler interrogation, and enlargement of the testis. In some cases of testicular torsion the intrascrotal portion of the edematous spermatic cord can be visualized at the upper pole [8]. Torsion is a critically important diagnosis as it mandates emergent surgical exploration. 

Epididymitis and epididymoorchitis are characterized by increased size and vascularity of the epidydimi with or without involvement of the testis. Torsion of the testicular or epididymal appendages can be seen as a characteristic round extratesticular nodule, frequently accompanied by scrotal skin thickening or hydrocele [9].

Lymphatic malformations of the scrotum are characterized as multicystic, extratesticular masses with moving internal echoes on ultrasound. The internal septa may show vascular flow on color Doppler [10].

AISE is a self-limiting condition, which tends to resolve in 3–5 days. NSAIDs and antibiotics have been used in management. A correct diagnosis can avoid surgical intervention and the characteristic ultrasound findings can be particularly helpful given the overlap in clinical presentation with testicular torsion and other conditions. The use of Doppler ultrasound was shown to reduce the rate of surgical exploration by more than half in one study [11]. The importance of good ultrasound technique is highlighted by this case. It can be difficult to demonstrate vascularity in the prepubescent testis on ultrasound. A high frequency transducer should be used and the scrotum should be scanned in both transverse and longitudinal section. The parameters for color Doppler imaging must be optimized for detection of low velocities and the color box should be sized and centered appropriately when assessing the testis. The use of pulsed-wave Doppler and power Doppler can be useful in challenging cases [8].

## 4. Conclusion

In conclusion, AISE is a rare cause of acute scrotum but important to recognize as it is a benign, self-limiting condition. The characteristic findings on ultrasound are those of edematous thickening and increased vascularity of the scrotal wall which produces the “Fountain Sign” on transverse color Doppler imaging. The testes and epididymis are normal in appearance and enlarged, hypervascular inguinal lymph nodes may be seen. Correct diagnosis can prevent unnecessary surgical exploration.

## References

[1] Abul F, Al-Sayer H, Arun N (2005). The acute scrotum: a review of 40 cases. *Medical Principles and Practice*.

[2] Qvist O (1956). Swelling of the scrotum in infants and children, and non-specific epididymitis: a study of 158 cases. *Acta Chirurgica Scandinavica*.

[3] Lee A, Park SJ, Lee HK, Hong HS, Lee BH, Kim DH (2009). Acute idiopathic scrotal edema: ultrasonographic findings at an emergency unit. *European Radiology*.

[4] Najmaldin A, Burge DM (1987). Acute idiopathic scrotal oedema: incidence, manifestations and aetiology. *British Journal of Surgery*.

[5] Van Langen AMM, Gal S, Hulsmann AR, De Nef JJEM (2001). Acute idiopathic scrotal oedema: four cases and a short review. *European Journal of Pediatrics*.

[6] Klin B, Lotan G, Efrati Y, Zlotkevich L, Strauss S (2002). Acute idiopathic scrotal edema in children—revisited. *Journal of Pediatric Surgery*.

[7] Geiger J, Epelman M, Darge K (2010). The fountain sign: a novel color doppler sonographic finding for the diagnosis of acute idiopathic scrotal edema. *Journal of Ultrasound in Medicine*.

[8] Aso C, Enríquez G, Fité M (2005). Gray-scale and color Doppler sonography of scrotal disorders in children: an update. *Radiographics*.

[9] Shalaby-Rana E, Lowe LH, Blask AN, Majd M (1997). Imaging in pediatric urology. *Pediatric Clinics of North America*.

[10] Morani AC, Ramani NS (2010). Lymphatic malformation in the scrotum. *Pediatric Radiology*.

[11] Varga J, Zivkovic D, Grebeldinger S, Somer D (2007). Acute scrotal pain in children—ten years' experience. *Urologia Internationalis*.

